# The Humble Charisma of a White-Dressed Man in a Desert Place: Pope Francis’ Communicative Style in the Covid-19 Pandemic

**DOI:** 10.3389/fpsyg.2021.683259

**Published:** 2021-09-01

**Authors:** Rosa Scardigno, Concetta Papapicco, Valentina Luccarelli, Altomare Enza Zagaria, Giuseppe Mininni, Francesca D’Errico

**Affiliations:** Department of Educational Sciences, Psychology, Communication, University of Bari “A. Moro”, Bari, Italy

**Keywords:** COVID-19, charismatic leadership, humbleness, multimodal signals, discourse analysis, we-ness, Pope Francis

## Abstract

The context of deep uncertainty, fear, and “social distancing” characterizing the COVID-19 pandemic has led to a need for cultural anchorages and charismatic leaders who may conjointly and effectively support human beings, strengthen their identity, and empower social commitment. In this perspective, the charismatic leadership of Pope Francis, which is widely shared not only within the religious world, may play a crucial role in facing emergency with existential reasons and psychological resources. The general aim of this work is to shed light on the communicative features of the charismatic leadership of Pope Francis during the pandemic emergency; in order to better understand his effectiveness, we analyzed both the core issues and his multimodal body signals in the global TV event of the Universal Prayer with the *Urbi et Orbi* Blessing. The multimodal and discursive analyses of the homily enabled us to define the “humble” charisma of the Pope, which is based upon on authentic and informal presence, manifested emotional signals (and, in particular commotion) showing features of equity and familiarity. From a discursive point of view, the common and overarching affiliation is constructed through a multiple focus on the “we” pronoun, which is constructed through socio-epistemic rhetoric. The results show how this integrated methodological perspectives, which is multimodal and discursive, may offer meaningful pathways detection of effective and persuasive signals.

## Introduction

The coronavirus epidemic was declared “COVID-19 pandemic” by the World Health Organization in March 2020. As a serious global health emergency, the main effective countermeasure across the first year was “social distancing” undermining the sense of being part of a community. If we consider the COVID-19 pandemic as a kind of watershed in the modern history of humanity, the acronym “B.C.” (before Christ), used in the Western world to indicate the whole period preceding the birth of Christ, could re-semanticized to designate the human condition “before COVID-19.”

Among the several subjective resources and cultural anchorages, religious forms of life (Belzen, 2005) can support believers in facing this unprecedented scenario. As opposed to philosophical and sociological claims about modernization and secularization, the essential role of religiosity in human life persists, as can be shown by the percentage of the world population still believing in God and by the strengthening role of religion within some societies (Bentzen, 2020). If in ordinary time, religions act as powerful “systems of meaning” (Park, 2010) addressing ultimate issues and concerns, in times of crisis human beings can witness a “turn” to religious contents and practices for comfort and explanations. As a matter of fact, almost all countries showed significant rises in prayer search; in March 2020, one of the most widespread kinds of prayer was the Coronavirus one, by means of which people asked for protection and strength and offered gratitude (Bentzen, 2020). A more general sign of the relation between the pandemic global fear and the religious “refuge” can be found in the considerable rise of a spiritual search (Maccioni, 2020).

The “searching” pathways can be empowered by the presence of charismatic leaders, since they concur to define and maintain social identity for their group (Hogg, 2001) and provide direction during times of uncertainty (Rast, 2015). In this perspective, the charismatic leadership of the Pope can play a crucial role in supporting prayers and in facing emergency with existential reasons and psychological resources. If we asked people to recollect the most significant and touching public images relating to COVID-19, probably most of them all over the world would talk about a lonely white-dressed man walking under the rain in a large empty square of the Eternal City. They would talk about Pope Francis celebrating the Universal Prayer with the *Urbi et Orbi* Blessing on March 27th. The enormous scope of this media event inspired social scholars and leaders with insights about the role of religious beliefs and practices, both in ordinary and extraordinary life events.

A recent study defined Pope Francis as a “digital leader” (Narbona, 2016), demonstrating the use that he made of his Twitter account for different areas, not only for the transmission of Christian values, but also for the proposal of specific actions and the fulfilling of more general educational tasks to both Catholic/Christian believers and people in general.

How is the leadership of the Pope characterized “offline” and how is it described in the COVID time? The present work aims to address some issues about communicative (both linguistic and bodily) features of the charismatic leader of the Christian Church during the pandemic emergency.

## The Effective Leadership: Charisma of Mind and Body

The socio-psychological literature assumes that charismatic leadership can be communicated by means of discursive practices (Seyranian and Bligh, 2008; Bligh and Kohles, 2009; de Vries et al., 2010) and non-verbal signals (Atkinson, 1984; Rosenberg and Hirschberg, 2009), pointing out that we can acknowledge a charisma of the mind and a charisma of the body (D’Errico et al., 2013).

As for the “charisma of mind,” recent literature outlines alternative approaches to the more traditional “all-about-me” studies; in line with the “New Psychology of Leadership” (Haslam et al., 2011), an effective leadership concerns leaders and followers as joining the same membership and fulfilling the same needs. In the more general idea of leadership as a process rather than as an individual property, this group process is grounded in a shared social identity or “we-ness,” emphasizing the psychological bond and the emotional leader–followers connection (Parry et al., 2019). This means that the effective leaders should encourage the collective sense of “who we are” and “what we are about” (Haslam and Reicher, 2016). Being both shared identity and shared reality at stake, the leader can gain influence by “shaping what we believe, what we value and how we should act” (Reicher et al., 2018, 129). In line with this model, a very recent review argues that instances of success and failure by different leaders during the pandemic are due to their ability to embody the shared interests of groups (Haslam et al., 2021).

The “charisma of the body” can be seen as a set of internal features of a person that, when manifested by some external displays, can influence people by inducing them to pursue some goals while feeling involvement and enthusiasm (D’Errico et al., 2013). Understanding charisma therefore means, on the one hand, to track the external displays in a multimodal perspective (Poggi, 2007) by analyzing words, prosody, voice, gesture, posture, face, gaze, body, and by decoding the expressed internal features.

The internal features expressed by the charismatic speaker can be potentially three: benevolence—caring for interests of others; competence—own expertise of an individual, planning capacity, and creativity; and dominance—the way to express own status position of an individual during the communicative event (D’Errico et al., 2013). In particular, the positioning of a speaker can be “vertical” and then communicate that she or he has more power/status than the others by means of several strategies (Poggi et al., 2011; D’Errico and Poggi, 2012); it can also communicate a horizontal/proximal stance toward the interlocutor by expressing on the contrary his/her own “humility” (D’Errico and Poggi, 2019). Although in the former case the speaker can raise the voice, interrupt, or overlap the interlocutor, in the latter case she or he can use longer pauses, lower intonation, and only occasionally incur in speech overlap, since she/he is awaiting his/her own turn to speak (D’Errico et al., 2019). They often look downward, with restrained gesture and tend to express negative emotions—especially sadness—while communicating their concern to the other (D’Errico, 2019). Generally speaking, humble stance is a “realistic” approach implying the awareness of being fallacious and imperfect. Despite humility being rather neglected in leadership literature (Collins, 2001), it is a fundamental “virtue of the heart”; more recently, it has been identified as an essential requirement of charismatic leadership, since it gives leaders a “charismatic touch” (Juurikkala, 2012). As a matter of this fact, in line with the Elicit–Channel (EC) model of charismatic leadership (Sy et al., 2018), leaders can both produce highly inspiring emotions from their followers and turn those emotions in actions that, if successful, result in positive outcomes, such as positive affect and trust.

This attitude implies several ones, such as (a) neglecting relevance to any external ornament like status symbols (Essentiality); (b) proposing a feeling/communication of equality to others (Equality feature); and (c) neither feeling nor displaying any superiority (Non-Superiority feature).

## The Research

### Aims and Research Questions

Within this framework, the overall goal of the present study is to investigate the charismatic leadership of Pope Francis through his multimodal communication. In particular, we tried to investigate his role of (religious) leader capable of harmonizing “contents” and “Gestalt,” and to identify his communicative features that help audiences cope with the COVID-19 emergency period.

In this social scenario characterized by deep uncertainty and fear, we suppose that his charismatic leadership will be exercised by focusing on a core issue, that is a common higher-order belonging; despite his being the supreme spiritual head of Catholicism, feelings of brotherhood and equality can be widespread by claiming the uniqueness of God and the coincidence between “His people” and “Humanity.”

This topic can be communicated through effective and persuasive communication acted by converging multimodal signals: as for the “charisma of mind,” we expect that discursive practices will be oriented to emphasize the value of “brotherhood” as a shared horizon for the highlighted problems and for their solutions (Pope, 2020a); as for the “charisma of body,” we hypothesize that his multimodal communication will emphasize the benevolence and the closeness to sufferings of others (empathy). A charismatic mind and body will be characterized by a humble communicative style.

### Procedures and Data

The present qualitative study is based on the multimodal analysis of a unique event—that is the Universal Prayer with the *Urbi et Orbi* Blessing on March 27th—which was transmitted live all over the world. Although the whole liturgy lasted for about 1 h and 24 min, the homily took about 16 min. This part of the liturgy was transcribed and analyzed through a multimodal approach focusing on body feature annotation and on paper-and-pencil discourse analysis.

### Multimodal Communication Analysis

In multimodal communication analysis, the facial expressions, gestures, and posture of the Pope were taken into consideration. As for facial expressions, both the Facial Action Coding System (FACS) coding model (Ekman, 1993) and Action Units (AUs) detection were also used. The selected video was included in ELAN (Max Planck Institute for Psycholinguistics, Nijmegen; Wittenburg et al., 2006), that is, an assisted annotation software tool in which categories (called “tier”) were created corresponding to each non-verbal communication signal.

After the annotation process, the following decoding step was the attribution of meaning to each non-verbal communication signal. Therefore, multimodality derives from the set of meanings of both verbal and non-verbal signals.

Hence, the result of this meticulous encoding and decoding procedure is not only composed of non-verbal signals with their related meanings according to humility features—that is familiarity, empathy, non-superiority, informality, equality, and essentiality (D’Errico and Poggi, 2019)—but also of the exact timing and signal duration.

### Discourse Analysis

The homily of the Pope was analyzed by the means of diatextual analysis (Manuti et al., 2017), that is a type of critical discourse analysis focusing on the reciprocal co-construction of texts and contexts emerging from each discourse (Scardigno et al., 2019). In line with the overall aim of this work, we selected some specific diatextual markers mainly having to do with the argumentative-rhetoric construction of the homily. More specifically, we drew on “social-epistemic rhetoric” (Berlin, 1993), that is a discourse construction that permits a top-down reading of texts by grasping sense perspectives valid for specific positioning groups.

An overview of the data was obtained through a semiotic square (Greimas and Courtés, 1979): it is a semiotic device enabling researchers to show the oppositional logic concerning every concept. In particular, the discourse enunciative positions can be organized upon three axes: opposition (white vs. black), contradiction (white vs. non-white), and subopposition (non-white vs. non-black). As such, the semiotic square is the concrete sign of the dialog between discursive data and interpretative options acted by social researchers.

### Main Results Discourse Analysis

#### Multimodal Analysis

In the analyzed communicative event, the multimodal communication of Pope Francis revealed high coherence among its diverse signals: no inconsistencies among facial expressions, posture, and gestures were found. This could be interpreted as an authentic attitude and spontaneous emotional pattern, despite its being an “official” celebration.

**FIGURE 1:**
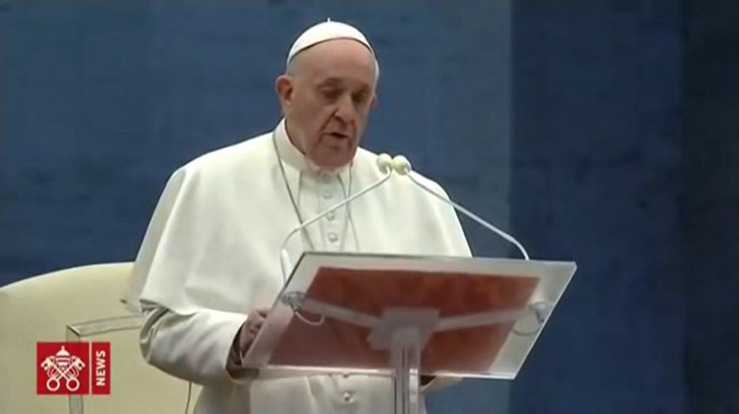
Pope Francis (minute 6.18).

An in-depth analysis across the minutes of the matching among signals and meanings enabled us to outline two essential features: (a) commotion as the main noticeable emotion and (b) the emphasis upon the verbal, through facial expressions and gestures. The average duration of the detected multimodal signals in this ceremony is 224 thousandths of a second out of 28 min of analyzed video. In the context of an official ceremony, the posture of the Pope is “open,” his shoulders turned to the audience that is ideally present, the ritual gestures are slow but as precise as the speech rate, he speaks in a low-pitched voice by clearly pronouncing the words. At the beginning of the homily, the Pope moves toward the altar, goes up and, with a trembling voice, and raises his eyes to heaven in order to give full sense to prayers and to ask for protection (“Through Christ our Lord”) (Figure 1).

**FIGURE 2:**
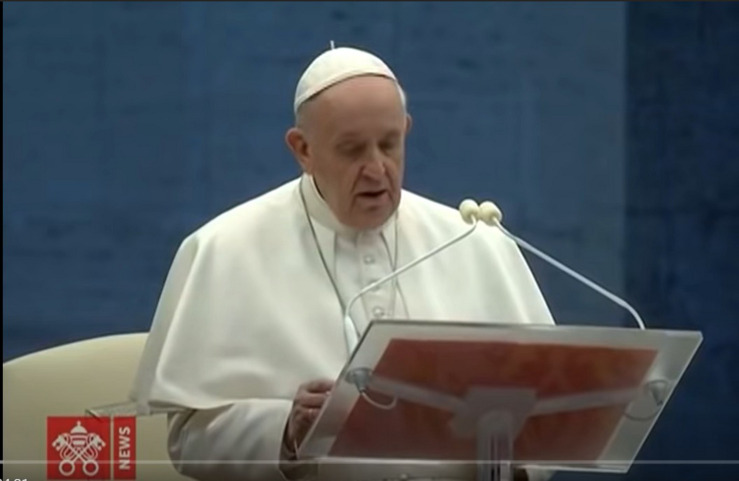
Pope Francis commotion by face (minute 10).

Such recurrent emotional signals as commotion follow sentences like “We found ourselves frightened and lost” (min. 10.58) expressing an emotional closeness to the suffering of other people. Figure 2 showed a significant example of commotion AUs detecting, such as AU15 + AU23 (Kimura et al., 2019): muscle contraction mainly involves the lips of the Pope. In particular, the outer part lowers and creates some sort of arc.

If the lip contraptions were decontextualized from speech and other signs, the identified AUs would be sadness. Nonetheless, in this case the movement of the head bends forward, not only to read the script, with a movement of 0.56 thousandths of a second. In addition, the corresponding verbal expression refers to the difficult period of the pandemic. As a consequence, the set of these multimodal signals can be holistically interpreted as “commotion”: the emotion of “movingness” is by definition included neither in joy nor in sadness; it rather introduces additional features found in highly selected episodes of sadness and joy (Kuehnast et al., 2014). An event of sadness arousing emotion is precisely the period of pandemic which aggravated uncertainty and fear during its first wave.

The moved and authentic emotion expression acted by the Pope represents a starting point to define him as a humble leader (Owens et al., 2019). The non-manipulation of emotionality, *albeit* in a formal ceremony, provides the audience with the image of someone feeling first man and then the Pope: in the guise of a man, he feels like the others and, in those of the Pope, he empathizes the mankind (*Equality feature*).

Features of “humility” are also outlined through the emphasis upon some discursive excerpts. In particular, batonic gestures (Navarretta, 2018)—for example movements of arms from the top to the bottom—are recurrent in the combination with expressions such as “We are.” The combined use of the first plural person and of the emphasizing batonic gestures (with one hand or both) empowers the feelings of *equality* and *familiarity*.

**FIGURE 3:**
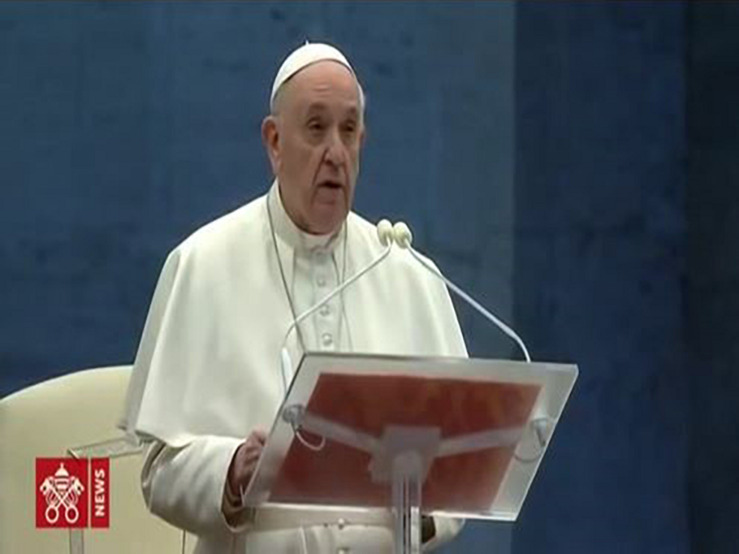
Emphatic facial expression (minute 15).

As for facial expressions, eyebrows contraction, especially in the inner part (AU4), can be observed (Figure 3). As already shown in Figure 1, this facial sign aims to strengthen and complete what is said (in this case “Always”).

In the central and final parts of the homily, the unit AU4 was found more frequently. Analyzing the verbal part being expressed synchronically with AU4 can help us to better understand the several meanings associated to this facial signal (Table 1); it can create a feeling of familiarity by emphasizing the sense of closeness (as in the case of “To enhance,” timing 00:25:08.709-00:25:08.929); otherwise, it can convey a mixture of empathy and slight anger by proposing a kind of “admonition” (as in the case of “The others say about us,” timing 00:20:09.205-00:20:09.425). Even in its variety, AU4 concurs to create the atmosphere of careful closeness, which strengthens the image of the Pope as a humble leader.

**TABLE 1:** Recurrence of AU4 emphasis expression and corresponding speech.

Timing	Speech
00:15:33.410-00:15:33.630	Pause
00:20:09.205-00:20:09.425	“The others say about us”
00:24:56.691-00:24:57.041	“Always”
00:25:08.709-00:25:08.929	“To enhance”
00:27:53.739-00:27:53.909	“To open spaces”

#### Discourse Analysis

In the particular scenario in which the mediatic event takes place, and in line with the multimodal communication of the Pope, discourse analysis reveals that his homily focuses on a really pathemic argumentation, where humility is mainly emphasized through equality attitudes.

Through a continuous contamination between the religious and secular domains, between “religionese” (Scardigno and Mininni, 2020)—that is the language of religion—and common speech, positive and negative connotations, “up” and “down” attitudes, what is at stake in this homily is a kind of “egalitarianism” overcoming religious borders and belongings, including all human beings. As a matter of fact, the Pope almost bans the “I” subject pronoun in favor of the first person plural, so that the whole discourse is dotted with “we”: subjects and verbs, adjectives, and pronouns testify to his commitment in the several experiences and connotations of human belonging.

**FIGURE 4:**
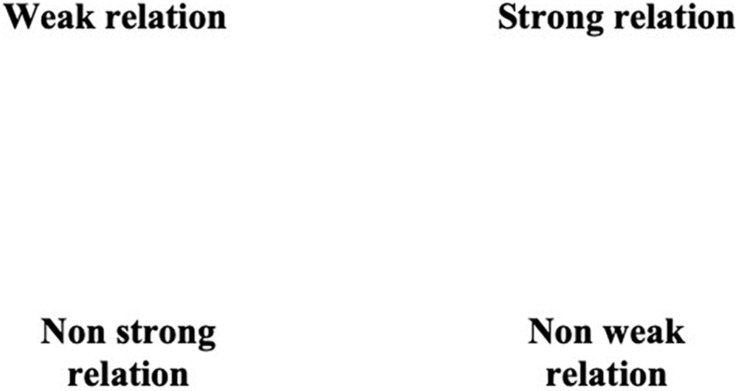
The semiotic square of “we” as styleme of humble communication.

In order to have both a synthetic and analytic vision of his “deep” discourse, a semiotic square was constructed (Figure 4). In line with the multiple dialectic and oppositive dimensions, we found the following oppositions:

(a) The basic opposition between “weak” and “strong” relations. Feelings, attitudes, and relations of human beings can be set in this basic opposition, concerning the nature of involvement in what is strictly “human.” Weak relations have to do with superficial attitudes and links, whereas strong relations with more careful and mindful ones.
(b) The consequent contradictions between “weak”/“non-weak” and “strong”/“non-strong” relations, involving different nuances of being involved and explaining the modulated “deepness” in constructing relations.
(c) The resulting subopposition between “non-weak” and “non-strong” relations, concerning the alternative quality of the nuanced activated relations.

The dilemmatic oppositions emerging from the semiotic square represent the axis upon which four types of “we” can be set:

(a) “weak”: when the Pope talks about this position and relations, he makes use of verbs and adjectives concerning human selfishness (e.g., “greedy for gain,” “we have not heard the cry of the poor”) and superficiality (e.g., “we let ourselves be absorbed by objects and stunned by hurry”), which are typical of the “Pre-COVID” time.
(b) “non-strong”: in this positioning, relations are founded on limited attitudes and exhibited through the unreadiness of humanity (e.g., “we found ourselves frightened and lost”) in facing with the global and social emergency during “COVID-time.”
(c) “strong”: this kind of “we” is elaborated through the discursive act of exhortation. As a matter of fact, in order to occupy this position, which highlights worthiness and emphasizes “solid relations”, the Pope aims for a “faithful” *we*, based on serious religious commitment:

“let’s invite Jesus in the boats of our life. Let’s give Him our fears, so that He can overcome them”.

(d) “non-weak”: as in type (c), this position can be set through the adhesion to more philanthropic proposals, which can transform “weak” relations in “non-weak” ones, aspiring to a “possible” *we*:

“[…] that common belonging from which we cannot escape, that is belonging as brothers.”

In such a multifaceted articulation, the Pope proposes a “religious” opposition between weak/sinful and strong/faithful *we* thus implying the “human” opposition between non-strong/unready and non-weak/possible *we*. In addition, the axis of weak/non-strong and strong/non-weak relations can be summarized and represented through two social-epistemic rhetorics, “fallibility” and “worthiness,” acting as frames for the opposite attitudes of common limitation, that is, faultiness, inadequacy, and imperfection of human beings, and full expression of human potential, based on the aspiration to goodness and rightness.

As for the pathemic connotation of discourse, the following additional discursive markers were found:

(a) *Metaphors*. In particular, three main kinds were used: (1) common speech metaphors, for example, “we went full speed,” referring to human and common habits; (2) religious metaphors, for example, “to embrace His Cross,” mainly deriving from the Christian symbolism; and (3) archetypal metaphors, for example, the “evening” representing the end of the day and life.
(b) Other *figures of speech*, such as climax, repetition, and the tripartite list:

“We have an anchor: in His Cross we were saved. We have a helm: though His Cross we were redeemed. We have a hope: in His Cross we were restored and embraced”.

These figures both creates an emotional atmosphere and improve the public involvement.

(c) *Polarized adjectives*, for example, “all of us fragile and disoriented but, at the same time, important and necessary.” The discursive politization acts as an additional emotional charge.

Both rhetoric figures and affective markers work in order to empower the empathic activation by increasing the perception of a strong relationship and of a humble leader.

## Discussion

The present qualitative analysis represented an attempt to investigate how the Pope proposed his undoubted charisma through a sensible mixture of discursive practices and multimodal signals making him a coherent and deep “living human document” (Ganzevoort, 1993) for an audience virtually coinciding with humankind, during an exceptional media event in the full pandemic emergency.

Since his first public appearance as a Pope, Jorge Mario Bergoglio characterized his communicative style through the “rhetoric of the unexpected.” Indeed, “the frame ‘first greeting of the Pope’ is reinvented—distances are shortened and hearts touched. From that day on, Pope Francis has often displayed unexpected behaviors” (Caffi, 2015, 23).

Since the “unexpected” magisterium and the pastoral action of Pope Bergoglio was also marked by the words “meeting” and “people” (Pope, 2020b), we supposed that in the selected event he would try to offer empowered pathways to highlight these concepts. The multimodal and discursive analysis of the homily enabled us to construct this type of frame; on the one hand, the work carried out with the ELAN-assisted annotation software tool revealed an authentic and informal presence, with manifested emotional signals, even in a formal celebration. The eyes, face, postures, and batonic gestures analyzed in their contexts are signals revealing both feelings of commotion, and equal, familiar, and emphatic stance. On the other hand, discourse analysis emphasized the performative strength of discursive practices in line with a pathemic argumentation based on metaphors and other rhetoric devices.

Common and overarching belonging is implemented through a multiple focus on “we,” which is constructed starting from the basic opposition between “weak” and “strong” relations. Looking at the four positions emerging from the semiotic square, a temporal axis can be outlined: while (a) is almost referred to the past and to the general attitudes/behaviors before the COVID-19 pandemic, (b) involves a particular actual time, that is the COVID-19 chronotope; both (c) and (d) imply a near-future time, that is welcome in line with the exhorted/proposed attitudes/behaviors. Nonetheless, the positions (c) and (d) are slightly different: the faithful *we* is exhorted on the basis of the confirmation of religious feelings and of the explicit presence of God in one’s own and common life; the possible *we* is founded on the (new) opportunity to construct worth and non-weak relations, that is on civic engagement and human care.

Taking into account the “twelve lessons” of the effective leadership associated with the crisis management proposed by Haslam et al. (2021), this work revealed the presence of several elements, such as the construction of a shared identity, the proposal of inclusive definition of in-groups, an empathic attitude, and so on. In a multifaceted and shared construction of human attitudes and behaviors, the Pope takes on his shoulders the responsibility of what is wrong and the opportunity of what can get better, thus defining at the same time not only “who we are” and “what we are about” (Haslam and Reicher, 2016), but first and foremost “what we can be” (the possible *we*) and “what we should be” (the faithful *we*).

Nonetheless, the multimodal communication of the Pope is unique in proposing a coherent model in the direction of humbleness. Confirming the unicity of “COVID-19 pandemic” as a kind of watershed in the modern history of humanity, both multimodal and discourse analyses of the homily of the “Urbi et Orbi” media event enabled us to consider the features of Pope Francis as a “humble leader”: his emphatic and moved attitude enhanced commitment; his charisma was specifically based on careful closeness and fraternal humanism and on an egalitarian contamination between religious and non-religious lexicon and belonging.

## Concluding Remarks

Since the charismatic leadership of Pope Francis is unquestionable both in the religious domain and in public opinion, the general aim of this work was to analyze how communication signals can play a significant role in His charisma, especially in such times of crisis and uncertainty as the COVID-19 pandemic. Social psychology of communication can offer a meaningful perspective for a conjoint focus on multimodal signals and discursive practices through the analysis of effective communicative performances. In order to better appreciate the several features of the charismatic leadership of Pope Francis, more extensive analysis on other communicative events should be carried out. Nonetheless, the proposed combined study can represent a novel methodological attempt to deeply analyze the associated meaning alongside linguistic and bodily signals. Specifically, the methodology used, which consists of multimodal and discursive analyses, can fall within the field of Social Signal Processing (Vinciarelli et al., 2011) and of Sentic Computing (Susanto et al., 2021). This effort has been applied also within the field of automatic signal detection, which recently have crossed both linguistic levels, as in the case of Sentiment Multimodal Analysis (Tiwari et al., 2020), and other body channel detection (e.g., facial or vocal expressions and body postures) (Soleymani et al., 2017). More in general, the recent remarkable development of the Affective Computing and the introduction of deep learning increased the development of more sophisticated systems (Wadawadagi and Pagi, 2020) aimed at predicting and automatically detecting emotional patterns starting from multimodal input (Morency et al., 2011; Poria et al., 2016; Chaturvedi et al., 2019; Yadav and Vishwakarma, 2019), with a real-time output (Cambria et al., 2013; Tran and Cambria, 2018). In this perspective, future studies will try to apply automatic systems to wider textual and visual corpora, promoting therefore interdisciplinary approaches.

## Data Availability Statement

The raw data supporting the conclusions of this article will be made available by the authors, without undue reservation.

## Ethics Statement

Written informed consent was obtained from the individual(s) for the publication of any potentially identifiable images or data included in this article.

## Author Contributions

RS as the first author, was responsible for the coordination of the whole project; in addition, she wrote a part of the introduction and of Sections “The Effective Leadership: Charisma of Mind and Body” and “The Research”; and she conducted and wrote the discourse analysis section and discussed its findings. CP conducted the multimodal communication analysis and reported results. VL wrote a part of Section “The Effective Leadership: Charisma of Mind and Body” and supported literature review; in addition, she took part in the discourse analysis phase. AZ took part in the discourse analysis process and wrote the concluding section of the manuscript. GM as a senior author, supervised the whole work; in addition, he wrote a part of the introduction and of Sections “The Effective Leadership: Charisma of Mind and Body” and “The Research”. FD’E supervised the theoretical, methodological, and analytical sections devoted to multimodal communication; in addition, she wrote part of Sections “The Effective Leadership: Charisma of Mind and Body” and “The Research”. All authors contributed to the article and approved the submitted version.

## Conflict of Interest

The authors declare that the research was conducted in the absence of any commercial or financial relationships that could be construed as a potential conflict of interest.

## Publisher’s Note

All claims expressed in this article are solely those of the authors and do not necessarily represent those of their affiliated organizations, or those of the publisher, the editors and the reviewers. Any product that may be evaluated in this article, or claim that may be made by its manufacturer, is not guaranteed or endorsed by the publisher.
